# Novel Electrohydraulic Technique of Cellulose Fiber Production from Industrial Hemp

**DOI:** 10.3390/polym17233178

**Published:** 2025-11-29

**Authors:** Undrakh Mishigdorzhiyn, Oksana Ayurova, Shunqi Mei, Belikto Tsydenov, Nikolay Ulakhanov, Kirill Demin, Yuri Grigoriev, Oyuna Tsybikova, Marina Namsaraeva

**Affiliations:** 1 Hubei Digital Textile Equipment Key Laboratory, Wuhan Textile University, Wuhan 430073, China; sqmei@wtu.edu.cn; 2 Institute of Physical Materials Science, The Siberian Branch of the Russian Academy of Sciences, Ulan-Ude 670047, Russia; nulahanov@mail.ru (N.U.);; 3 Department of Inorganic and Organic Chemistry, Banzarov Buryat State University, Ulan-Ude 670000, Russia; 4 Department of Law, Intellectual Property, and Forensic Expertise, Bauman Moscow State Technical University, Moscow 105005, Russia; elhydra@yandex.ru; 5 Department of Plant, Meadow, Fruit and Vegetable Growing, Buryat State Agricultural Academy Named After V.R. Filippov, Ulan-Ude 670034, Russia; oyuna_sodnom@rambler.ru (O.T.); nmmarina@mail.ru (M.N.)

**Keywords:** industrial hemp, electrohydraulic effect, delignification, hemp fiber, cellulose materials, biocomposites

## Abstract

The key stage of processing high-cellulose hemp raw materials is delignification—the removal of lignin and hemicelluloses to obtain strong cellulose fibers. This study experimentally demonstrated the effectiveness of using the electrohydraulic effect (EHE) to delignify high-cellulose hemp raw material, which can then be used as a base for composite materials. Hemp raw material, in the form of 50 mm-long straws, was placed in a water-filled chamber and exposed to a shock wave generated in the water by an electric discharge with an energy of 1.6 kJ at a voltage of 50 kV. The tensile strength of the treated fibers after combined processing (NaOH/EHE) and after EHE reached 262 MPa and 201 MPa, correspondingly, which are 5% and 37% higher than after chemical delignification in a NaOH medium (191 MPa). Cellulose materials obtained from cellulose fiber after EHE exhibit higher strength properties compared to materials based on cellulose obtained by delignification in a NaOH medium. Thus, the tensile strength (σ) of materials made from fibers after EHE was 4.37 MPa, after combined NaOH/EHE treatment 1.94 MPa, and after alkaline treatment 3.95 MPa. EHE reduced delignification time by 2–20 times compared to NaOH treatment and eliminates the need for an additional fiber separation procedure. The use of EHE is proposed as a highly cost-effective, technologically and environmentally sound solution for producing hemp fiber for use in biocomposites, woven, and non-woven materials.

## 1. Introduction

In the context of the global transition to sustainable materials, natural fibers, particularly those derived from industrial hemp, are emerging as a priority eco-friendly alternative to synthetic fibers in composites and functional textile materials. Their advantages include biodegradability, renewability, accessibility, and contribution to a low-carbon economy. The key stage in processing high-cellulose hemp raw materials (cellulose content ~70%) is effective delignification, which involves the removal of lignin and hemicelluloses to obtain strong cellulose fibers. The search for optimal pre-treatment methods remains highly relevant.

The electrohydraulic effect (EHE) was first discovered by the Soviet scientist L.A. Yutkin and involves converting electrical energy into mechanical energy by passing an electrical discharge through a liquid medium [1]. It is known that EHE initiated by a pulsed electric current discharge is referred to as pulsed arc electrohydraulic discharge (PAED) or electrical explosion shockwave technology [2,3]. The process of energy transfer to water occurs through a plasma channel formed by a high-current or high-voltage electric discharge between two electrodes immersed in water. Therefore, in the international literature, the term plasma pulse system (PPS) is also used to describe devices that generate a pulsed arc electrohydraulic discharge [4]. The method is applicable for grinding a wide range of difficult-to-process materials, such as rocks and fibers [5].

The essence of EHE is that a powerful electrical discharge in a liquid generates extremely high hydraulic pressure, which can exert substantial force. The high-density electric current results in a concentrated release of Joule heat, causing intense heating of the plasma. Without rapid heat removal, the gas temperature rises quickly, resulting in a rapid increase in pressure within the current channel, which initially has a small cross-sectional area. A cylindrical compression wave forms in the liquid due to the rapid expansion of a vapor-gas cavity driven by internal pressure. The intense energy release in the channel can cause its expansion velocity to exceed the speed of sound in the liquid, converting the compression pulse into a shock wave. The cavity’s volume continues to expand until its internal pressure drops below the external pressure, causing it to collapse. When the pressure drops, the material, compressed by the shock wave, straightens and breaks into fibers.

The productivity of such installations increases proportionally to the discharge energy and the discharge pulse repetition rate. That is, it can be varied over a wide range without altering the mechanical part, simply by increasing the power source. Experience in operating such installations in stone crushers has shown that, with an appropriate power source, stable operation is maintained at least up to a frequency of 4 Hz. Productivity increases proportionally to the frequency. Since the effective processing zone is relatively small due to shock wave attenuation, it is advisable to use multi-electrode circuits, as it was reported in [6]. EHE is suitable for hemp fiber delignification and its primary processing, eliminating the need for grinding raw materials and the use of chemical reagents.

Natural fibers are formed in natural conditions without direct anthropogenic intervention and are classified by origin into plant, animal, and mineral fibers [7]. Plant fibers are extracted from various parts of plants, such as seeds (cotton), stems (flax, jute, ramie), leaves (sisal, abaca), or fruits (koyr) [8]. Animal fibers include wool (from sheep, goats, and camels) and silk (produced by silkworms). Mineral fibers are obtained from rocks (basalt, quartz, glass, and asbestos) [7].

A common structural feature of many natural fibers (especially plant fibers) is the presence of a single strand consisting of cellulose microfibrils that are spirally oriented in the layers of the secondary wall around the central channel. The microfibrils, which have a crystalline structure, are embedded in an amorphous matrix that contains heteropolymers of lignin (an aromatic phenolic polymer) and hemicellulose (a heteropolymer of pentoses and hexoses) [9,10,11].

The main structural biopolymer in plant fibers is cellulose, a high-molecular-weight carbohydrate with a high degree of orientation. Its content in the cell wall varies depending on the plant species, growth conditions, and maturity stage [12]. The properties of fibers are directly affected by the nature of the raw material [13]. Plant fibers, often by-products of agriculture (primary) or processing (secondary), are mainly made of cellulose and typically exhibit high tensile strength [14]. However, their hydrophilic nature makes them prone to moisture absorption, which can cause defects in polymer matrices and significantly decrease the mechanical properties of composite materials. On the other hand, wetting fabrics made from hemp and cotton fibers can increase their tensile strength by up to 15%. Therefore, careful selection of natural fibers is essential in designing biocomposites [15].

The domestic pulp and paper industry relies primarily on wood raw materials and imported cotton fiber [16]. The country’s geoclimatic conditions significantly limit the possibility of large-scale cotton cultivation. Meanwhile, global demand for cotton has shown steady growth, accompanied by difficulties for key producers in maintaining stable supply volumes and the required quality characteristics of their products [17]. The situation with wood pulp suitable for the production of high-tech fibers (α-cellulose content ≥ 94%) is also characterized by Russia’s dependence on imports.

**Figure 1:**
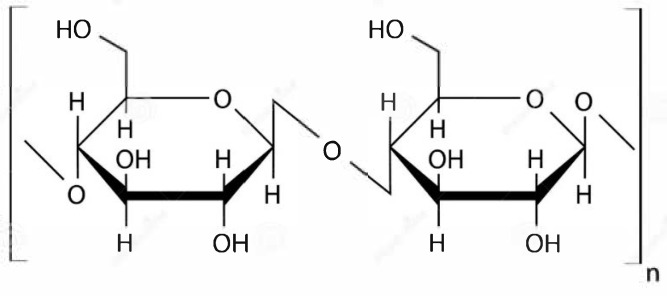
The structural formula of cellulose.

The cellulose content varies depending on the source: coniferous/deciduous wood (40–60%), reed/cane (up to 42%), corn straw/stems (~30%), hemp (70–75%), and cotton (92–95%) [18]. The chemical structure and supramolecular organization determine the physical and chemical properties. Cellulose is a linear homopolysaccharide consisting of β-D-glucopyranose units connected by β-1,4-glycosidic bonds (Figure 1). The properties of the polymer are strongly influenced by intermolecular interactions (hydrogen bonds and Van der Waals forces), whose total energy (cohesion energy) significantly exceeds the energy of covalent bonds in long macromolecules [19]. Hydrogen bonds (17–34 kJ/mol) formed between the hydroxyl groups of adjacent units and macromolecules provide chain rigidity in the solid state and dominate over Van der Waals forces (~4 kJ/mol).

The supramolecular structure of cellulose is characterized by the presence of crystalline and amorphous regions in the microfibrils. Crystallites (areas with long-range three-dimensional order and strong intermolecular interactions) provide strength. Amorphous regions (with short-range order) are more accessible to reagents and solvents, determining their reactivity. Natural cellulose has a crystallinity of 65–75% and an amorphinity of 25–35%. This heterogeneous structure (without a sharp phase boundary) is a key factor in determining the technological properties of cellulose [19].

One of the primary issues in pulp production is the high lignin content in the raw material, a component that is difficult to process. In wood, lignin levels can reach 40% [20]. Compared to wood resources, technical hemp has a significantly lower lignin content, ranging from 0% to 15% in the fibers and up to 30% in the bast (the woody parts of the stem). This notable difference in lignin content, especially in the valuable fibrous part of technical hemp, positions it as an environmentally friendly raw material for cellulose production, helping to reduce the amount of lignin waste that is hard to dispose of.

The choice of delignification method affects the amount of lignin and related substances removed, which impacts the properties of the resulting technical pulp. Chemical methods, based on the fact that lignin is broken down by many chemical reagents more quickly than cellulose, are labor-intensive, time-consuming, and not environment-friendly [21]. The chemical (alkaline) treatment is conducted in 5–14% NaOH solutions for 24 h at ambient temperature or for 0.75 to 4 h at 160–170 °C [22,23,24].

This study evaluates the efficiency of delignification using the EHE, which involves treating the processed material with a powerful electrical discharge in water, generating a shock wave. This approach is regarded as cost-effective (by removing the need for raw material grinding) and environment-friendly (by avoiding the use of chemical reagents). This allows the consideration of the EHE method as a technically and ecologically sustainable approach to tackling the challenge of hemp fiber delignification, enabling its use in high-performance composites. The operating principle of EHE will be described in more detail in the Methodology section.

## 2. Materials and Methods

The study was conducted on samples of technical hemp threshing (dry, decomposed stems) obtained from the Department of Plant, Meadow, Fruit and Vegetable Growing at the Buryat State Agricultural Academy named after V.R. Filippov [25]. The process of extracting bast fibers from the threshing was carried out using mechanical defibration according to the methodology described in [26].

**Figure 2:**
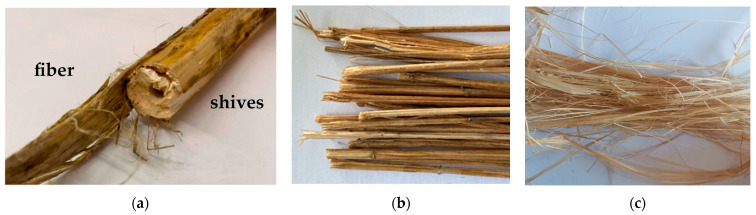
Hemp raw materials: retted (**a**,**b**) and bast fibers (**c**).

The raw materials used were hemp retted and bast fibers, each 50 mm in length (Figure 2). Retted fiber is flax or hemp straw that has been thermally, biologically, or chemically treated. Retted fiber contains cellulose in the fiber and shives. The latter is the woody part of the stems of spinning plants.

### 2.1. Chemical Delignification

Samples of hemp raw materials (bast fiber) were subjected to alkaline treatment in 500 mL of a 4% NaOH solution at 90 °C for 5 h with constant stirring. After the reaction was completed, the reaction mass was cooled and filtered under vacuum. The filtrate (leach) was collected for subsequent lignin extraction. The resulting precipitate was successively washed with distilled water until the pH of the filtrate reached 6–7, and then treated with a 2% solution of HNO_3_ (decationization) for one hour with stirring. The product was washed with water until the medium had a neutral pH and then it was squeezed [26,27].

### 2.2. EHE Delignification

**Figure 3:**
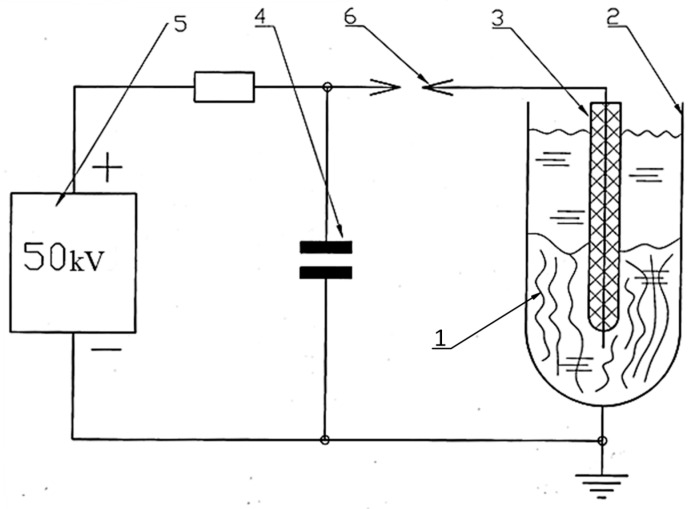
Schematic diagram of the electrohydraulic installation by Yu.V. Grigoriev, where **1** is the raw material, **2** is the water-filled steel chamber, **3** is the electrode, **4** is the capacitor, **5** is the high-voltage source and **6** is the spark gap.

**Figure 4:**
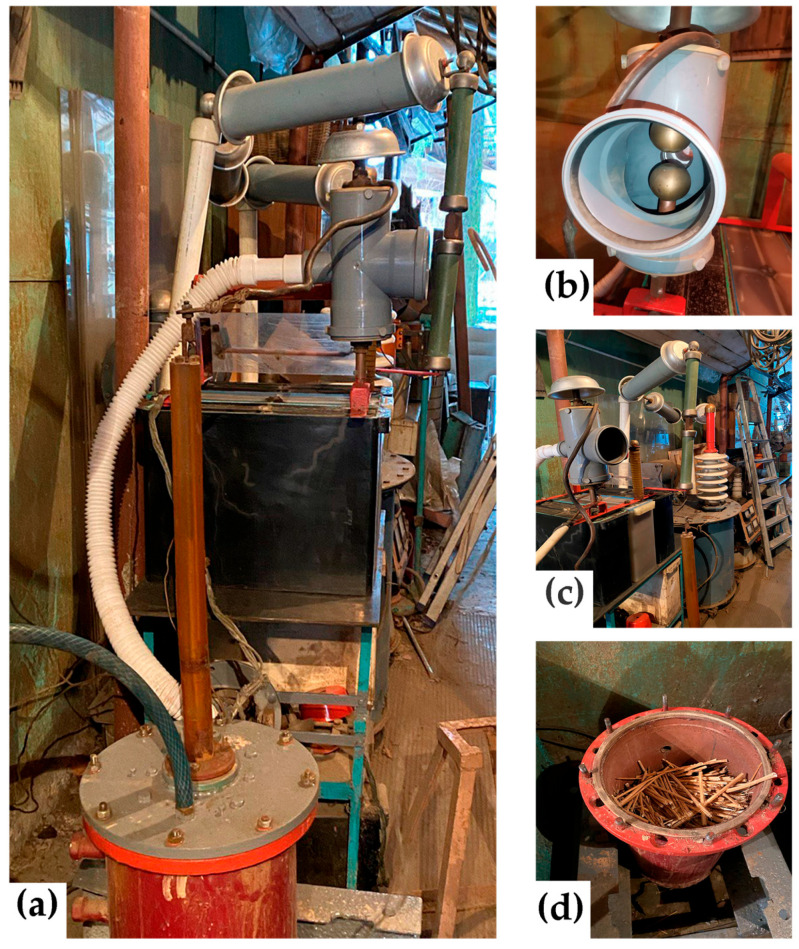
EHE installation, general view (**a**), spark gap (**b**), general view with a high voltage transformer (**c**) and discharge chamber with hemp retted fiber (**d**).

Delignification of hemp raw materials was carried out using an electrohydraulic unit developed by Yu.V. Grigoryev. The unit’s schematic is shown in Figure 3, and photographs of its individual components are shown in Figure 4.

From Figure 4, it is evident that electrohydraulic equipment is straightforward in its circuitry and design, both in production and control. The raw material *1* to be processed was placed in a water-filled steel chamber *2* and subjected to a shock wave and cavitation generated by a powerful electric discharge between electrode *3* and the bottom of chamber *2*. The discharge occurred when the voltage on capacitor *4*, charged by high-voltage source *5*, reached the breakdown voltage of spark gap *6*. After the discharge was complete and the spark gap’s electrical strength had been restored, the capacitor was recharged, and the cycle was repeated. The discharge voltage can be adjusted by varying the distance between the spark gap electrodes (Figure 4b), and the discharge power can be controlled by changing the discharge voltage or the capacitance of capacitor *4*.

During the experiments, the discharge energy in the setup was 1.6 kJ at a voltage of 50 kV. The discharge pulse duration was 10 μs. Due to the low power of the power source, the discharge pulse repetition rate was 10 discharges per minute. The processing time in this mode was 15, 40 and 50 s. The initial pH of the working fluid is 7.4.

In addition, the samples underwent a combined treatment, where they were first subjected to alkaline delignification for 2 h, followed by EHE treatment for 15 min. All delignification modes are presented in Table 1.

### 2.3. Physical and Chemical Characterization of Hemp Materials

**Figure 5:**
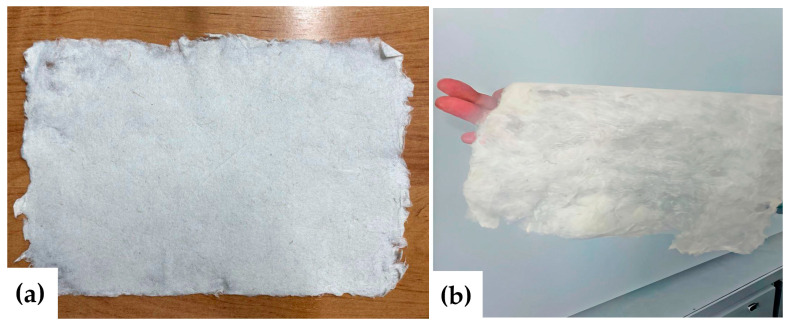
The cellulose material obtained from hemp fibers on a flat surface (**a**) and on the palm (**b**).

The processed cellulose fibers were ground in an Altimax laboratory mill (Changzhou Xingyun Electronic Equipment Co., Ltd., Changzhou, China) for 30 min. The prepared 6%-cellulose pulp was placed in a mold with a perforated bottom and rolled with a roller. The cellulose material, in the form of a wet sheet, was subjected to hot pressing and then dried at 100 °C for 5 min (Figure 5) [28].

**Figure 6:**
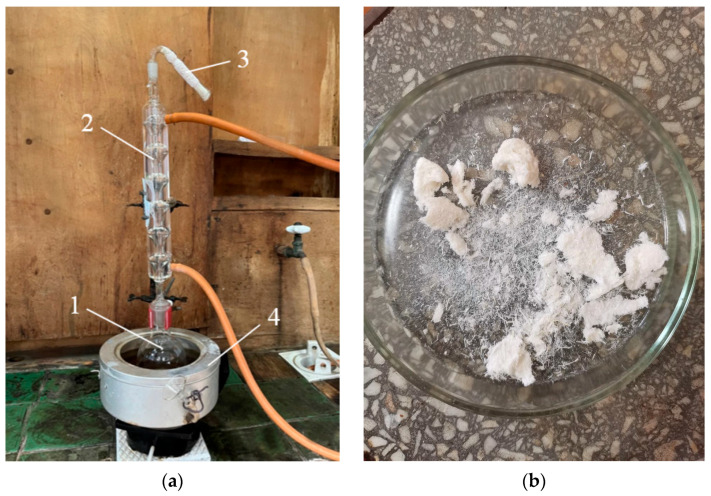
Apparatus for determining cellulose content (**a**), where 1—round-bottomed flask, 2—reflux condenser, 3—calcium chloride tube, 4—water bath. Cellulose after nitric acid hydrolysis (**b**).

The cellulose content of the target product was determined by nitric acid hydrolysis in an ethanol medium using the apparatus (Figure 6) [26,27]. A sample was sequentially treated with a mixture of concentrated nitric acid and ethanol. Ethanol inhibits the degradation of cellulose by the acid, while hemicelluloses are predominantly hydrolyzed. Lignin undergoes nitration and partial oxidation, dissolving in the solution. After four treatment cycles, the cellulose became discolored, acquiring a pure white color (Figure 6b).

XRD analysis of the samples was performed on a D2 PHASER powder diffractometer (Bruker AXS GmbH, Karlsruhe, Germany) using CuKα radiation in the 2θ range of 10–70° at room temperature at the Materials Science Laboratory, Dorzhi Banzarov Buryat State University. The degree of crystallinity (*CrI*) of the hemp fiber was calculated by the Sigal Empirical Method:

$$Crl\;\left(\%\right)=\frac{I_{\left(200\right)}-I_{am}}{I_{\left(200\right)}}\cdot 100$$

where

*I*_(200)_—Net intensity of the peak (200);

*I_am_*—Intensity of the amorphous background at ~18° (between (1̅10) and (200)) [29].

**Figure 7:**
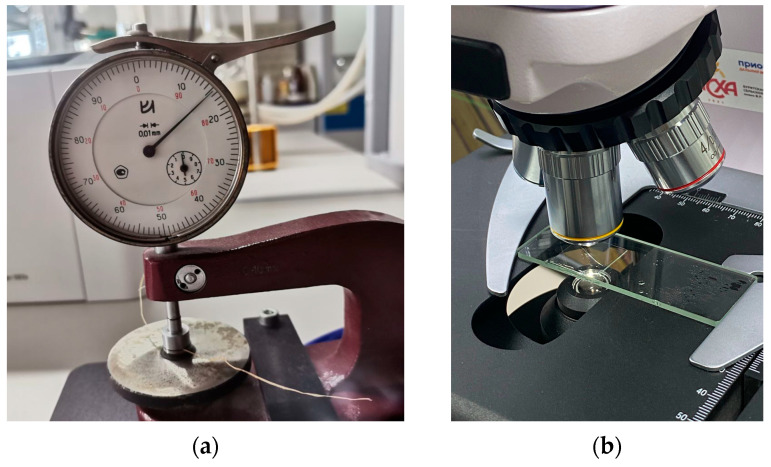
Measurement of the diameter of a cellulose fiber sample, using a thickness gauge (**a**) and using an optical microscope (**b**).

The diameter of the cellulose fibers was measured using a TN 10–60 thickness gauge (PLC NPO KirovInstrument, Kirov, Russia), with the average value recorded from three measurements taken at different points on the sample (Figure 7a). The measurement error was less than 0.01 mm.

The cross-sectional dimensions of each fiber within the working area were further monitored using a Magus Bio 230TL optical microscope equipped with a Magus CHD 10 digital camera (PJSC Levenguk, St. Petersburg, Russia) at the Laboratory of Medicinal Herbs, V.R. Filippov Buryat State Agricultural Academy. Three measurement points were randomly selected for each image analysis, and the final diameter was determined as the arithmetic mean (Figure 7b).

Mechanical tensile tests of cellulose fibers and composite materials based on them were carried out according to the standards ASTM D 3379-75 and GOST 13525.1-79 [30,31,32]. The length of the fiber samples was 25 mm, and the length of the material samples was in the form of strips measuring 15 × 100 mm. The tests were performed on an IR 5092-5.5 tensile machine (LLC Impuls, Ivanovo, Russia) at the Interdepartmental laboratory of the V.R. Filippov Buryat State Agricultural Academy. The dependence of tensile stress on deformation was calculated, and the modulus of elasticity, tensile strength, and relative elongation at break were determined. The results were averaged based on 10 measurements for cellulose fibers and on 6 measurements for composite materials for each sample type according to the standard [26].

**Figure 8:**
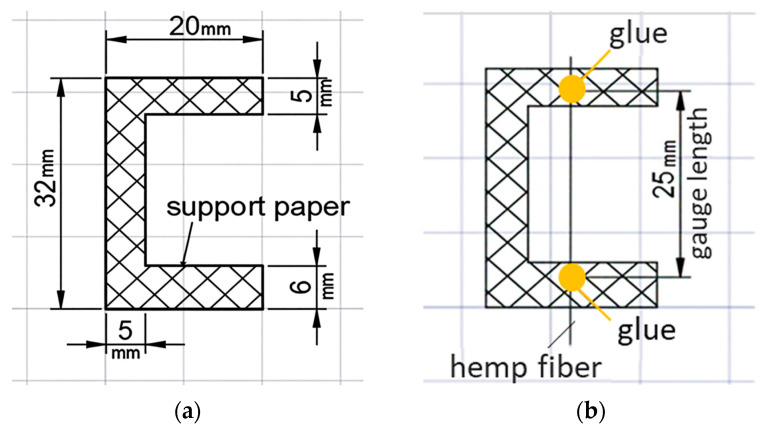

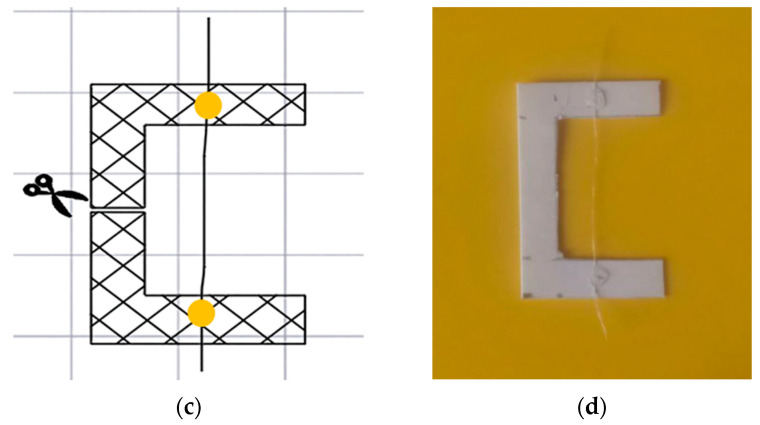
Scheme for preparing a cellulose fiber sample for tensile testing.

Samples of individual fibers were fixed for testing using the paper frame method, where the fibers were secured on a thick paper base with adhesive (Figure 8).

**Figure 9:**
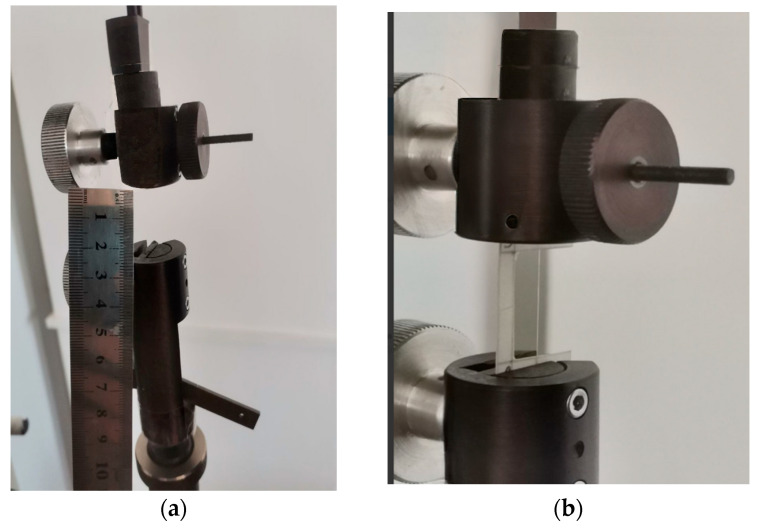
Distance between the grippers (measuring length) (**a**); installed sample of cellulose fiber in the grippers (**b**).

Key mechanical parameters (tensile strength and elongation at break) were calculated based on force and displacement data recorded by the testing machine (Figure 9). The calculations used the initial cross-sectional area and initial length of the sample. Test conditions were as follows: tensile speed of 0.1 mm/min, preload of 1 N, and preload application speed of 20 mm/min.

The adsorption capacity of cellulose materials was evaluated in glass flasks on a THYS2 shaker (VEB Medizintechnik (MLW), Leipzig, Germany) until equilibrium was reached (24 h). A 0.01 g sample was contacted with 10 mL of a dye solution (100 mg/L). The adsorption properties of cellulose materials for cationic dyes—methylene blue (MB) and crystal violet (CV)—were assessed. The dye concentration after adsorption was measured spectrophotometrically at wavelengths corresponding to the absorption maximum: 667 nm for methylene blue and 590 nm for crystal violet, using previously constructed calibration curves.

Adsorption efficiency (E, %) was determined by the formula:

(1) $$E\;=\;\frac{C_{0}-C_{24}}{C_{0}}\cdot 100\%,$$

where C_0_ is the initial concentration of the dye solution, mg/L, and C_24_ is the concentration of the dye in the solution after 24 h [33].

The specific surface area of the obtained materials was determined using the low-temperature nitrogen adsorption method, as described by the Brunauer–Emmett–Teller (BET) theory, on a ThermoSorb LP apparatus (LLC Katakon, Novosibirsk, Russia). Samples with identical mass were taken to improve the reproducibility of the specific surface area analysis data. The sample was subjected to thermal conditioning at 60 °C for 120 min to remove various gases. After this, the samples were cooled to 25 °C and analyzed using the mentioned apparatus.

## 3. Results

**Figure 10:**
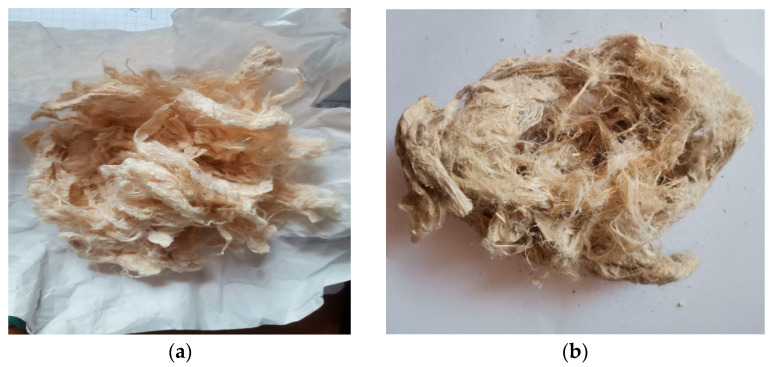
Cellulose fiber after alkaline (**a**) and after EHE (**b**) delignification.

Figure 10 illustrates the visual differences between the cellulose products obtained using the alkaline and EHE methods. The observed difference in coloration between the samples is likely due to the residual lignin content, with the most intense color corresponding to the sample with the highest lignin content [27].

The cellulose content in the delignified fiber was determined using the Küschner method [26,27]. The study’s results are presented in Table 2. The cellulose product obtained using the EHE method contains less cellulose (60.7%) and has a darker color compared to the product obtained using the combined method (Figure 10b).

### 3.1. The Effect of Fiber Diameter on Its Strength Characteristics

**Figure 11:**
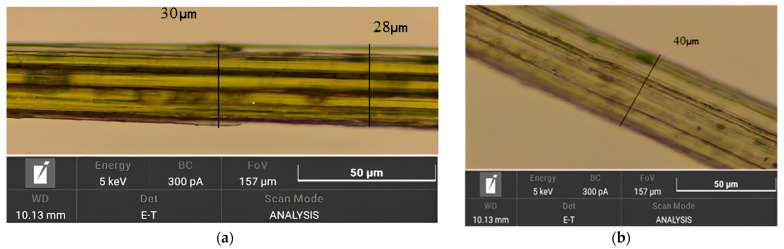
Microimage of cellulose fiber after alkaline (**a**) and after EHE (**b**) delignification.

To evaluate the functional properties of the materials, their mechanical characteristics were studied. Natural fibers (unlike synthetic ones) are morphologically heterogeneous and sensitive to external factors [34]. Fiber diameter is critical for strength and elastic modulus, so it was measured with a thickness gauge and microscopy. As a result, the following points were established:

1. Thinner fibers exhibit increased strength (likely due to fewer defects);
2. Optical microscopy revealed a bundle-like structure of fibers instead of individual fibrils (Figure 11);
3. Morphological heterogeneity and differences in fiber diameter explain the significant variation in strength from 55 to 353 MPa (Appendix A, Table A1) in samples after 40 min of electrohydraulic treatment.

**Figure 12:**
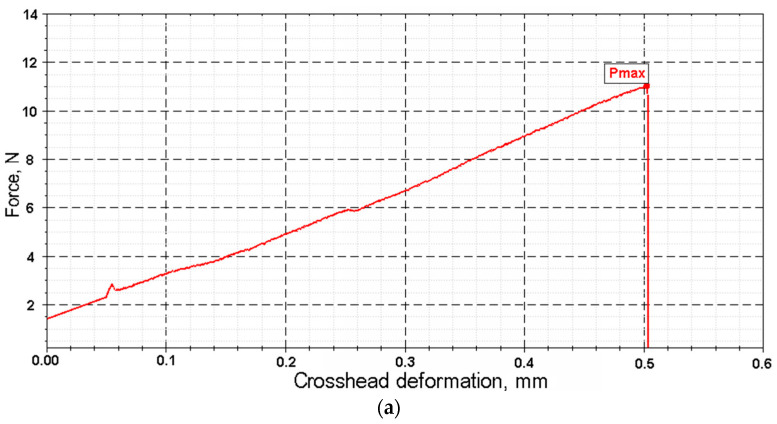

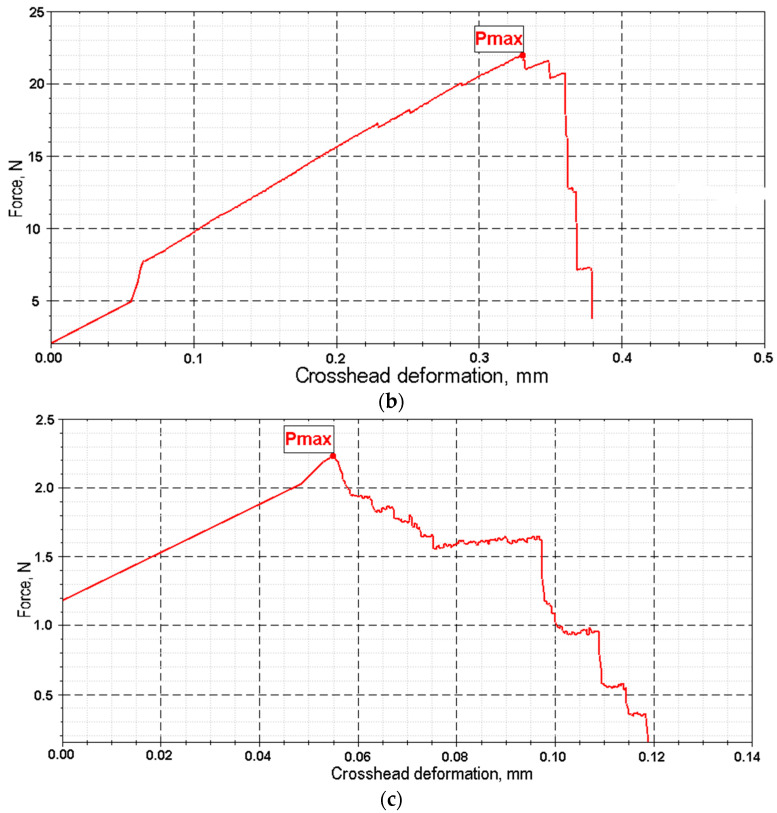
Graph of stress–strain dependence for cellulose fiber in different diameter ranges: (**a**) d < 50 μm; (**b**) 50 μm < d < 100 μm; (**c**) d > 100 μm.

As shown in Figure 12, fibers with a diameter of less than 50 µm exhibit a fundamentally different deformation mechanism. Their stress–strain curves combine linear elasticity with extreme values of Young’s modulus and a subsequent strengthening plateau, which correlates with record strength (Table A1) [30]. This effect is attributed to the reorientation of fibrillar elements with a minimal defect density.

**Figure 13:**
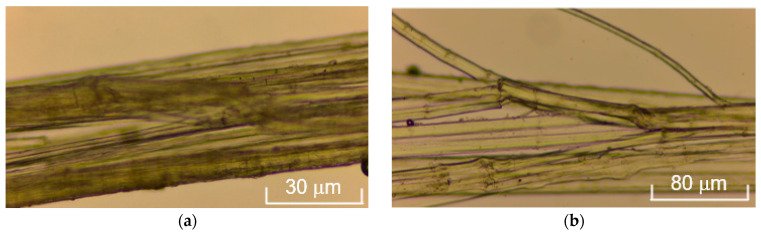

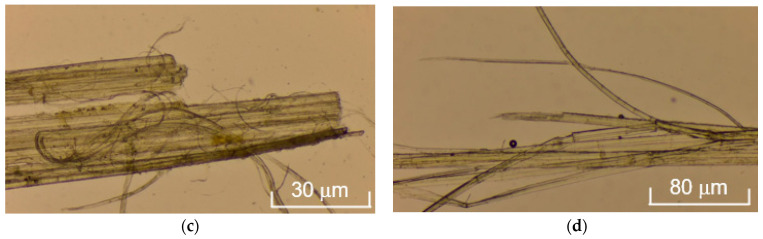
Microimages of cellulose fiber samples after tensile strength testing: fiber section with delamination at magnification ×40 (**a**), fiber section with delamination and rupture of individual microfibers at magnification ×100 (**b**), rupture of microfibers at magnification ×40 (**c**) and ×100 (**d**).

The decrease in the strength of large-diameter fibers is attributed to the accumulation of structural imperfections (microdestruction, dislocation, and disorientation of fibrils) and the weakening of interfibrillar boundaries (Figure 13).

**Figure 14:**
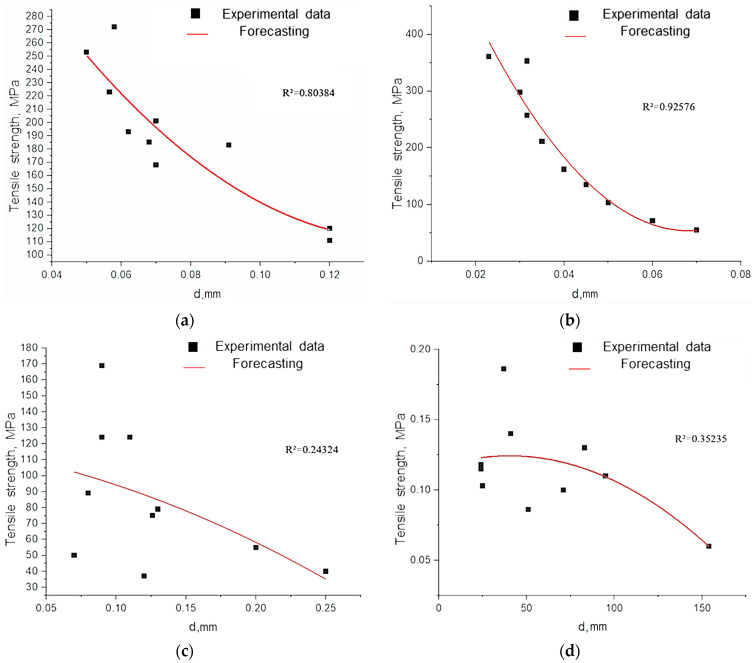

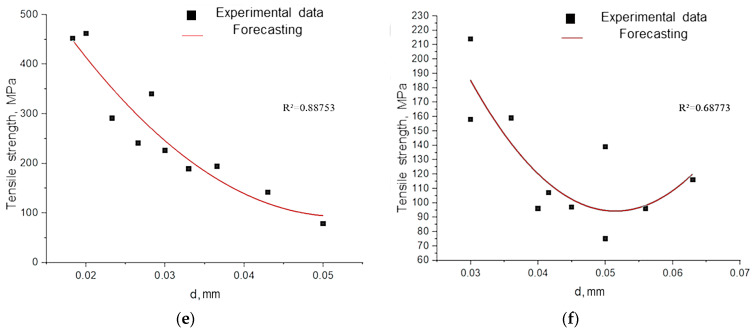
Tensile strength of cellulose fibers depending on their diameter: (**a**) untreated hemp fiber; (**b**) cellulose fiber (EHE for 40 min); (**c**) cellulose fiber (EHE for 15 min); (**d**) cellulose fiber (EHE for 50 min); (**e**) cellulose fiber (NaOH/EHE); (**f**) cellulose fiber (NaOH, 2 h).

The experimentally established inverse correlation between fiber diameter and strength has a fundamental basis: the probability of defect formation in heterogeneous biopolymer systems increases proportionally to the volume of the material (Figure 14). Our observations are consistent with the Griffiths critical defect model, which suggests that larger fibers contain more fracture-initiating heterogeneities.

### 3.2. Influence of the Type of Processing on the Strength Properties of Cellulose Fibers

Average values of deformation-strength properties of cellulose fibers, which determine the strength of cellulose fibers (Table 3). EHE treatment of retted fiber for 40 min provides an average value of tensile strength (σ) of the fiber of 201.0 MPa. The maximum value (262.0 MPa) corresponds to the combined treatment (NaOH/EHE for 15 min) of bast fibers. These modes are characterized by maximum values of Young’s modulus of 50.4 and 75 GPa, respectively. The increase in density is associated with an increase in cellulose crystallinity, resulting from the removal of lignin and hemicellulose, which in turn leads to a decrease in the mobility of macromolecular chains [32]. This is confirmed by the values of relative elongation, where the minimum values are characteristic of the mentioned modes.

**Figure 15:**
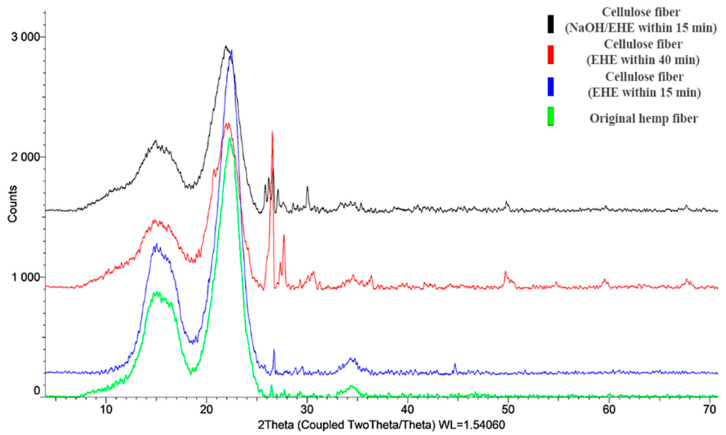
XRD patterns of cellulose fibers before and after delignification.

XRD analysis confirms the assumption that the increase in crystallinity after EHE is greater than that achieved by the chemical method. After delignification, characteristic reflections of the crystalline phase of cellulose are observed in the XRD patterns at 2θ = 14–16° for the triclinic form and in the range of angles of 22–24° for the monoclinic form (Figure 15), which is consistent with reference [32]. Comparative analysis of the experimental data showed that in all cellulose samples, the main crystalline phase is the crystalline monoclinic form of natural cellulose (according to the ICDD database (PDF-2). The reflections characteristic of the triclinic form shift towards smaller angles, indicating an increase in the interplanar distances between the cellulose chains, as a result of the combined treatment (NaOH/EHE) and EHE treatment for 40 min [35,36]. This is explained by the functionalization of the polymer chains with chemical groups separating the planes. The intensity of the reflections varies depending on the treatment method, confirming the correlation between crystallinity and strength properties.

The XRD patterns of all hemp fiber samples demonstrate characteristic reflections of cellulose Iβ: a broad peak at 2θ ≈ 15° (superposition of the (1̅10) and (110) planes, d ≈ 5.9–6.0 Å), an intense peak at 2θ ≈ 22° (the (200) plane, d ≈ 4.0 Å), and a weak reflection at 2θ ≈ 34.5° (the (004) plane, d ≈ 2.6 Å). The absence of the peak at ~20° and the preservation of the reflection at 22.5° indicate the conservation of the native structure without transition to cellulose II.

The degree of crystallinity calculated using the Segal method is 80.2% for the untreated fiber. The EHE treatment for 40 min and NaOH/EHE treatment increase crystallinity to 83.0% and 83.5%, respectively. This indicates the removal of amorphous components (hemicellulose, lignin) and ordering of the crystalline phase under the influence of EHE.

The study of mechanical and structural properties of hemp fibers demonstrated that the delignification of hemp raw materials by EHE can be carried out 2–20 times faster than by the chemical method, thereby bypassing the stage of manually or mechanically separating fibers from shives on mashing and threshing machines. Moreover, the resulting cellulose fibers after EHE and combined treatment (NaOH/EHE) exhibit greater strength characteristics than those obtained by the traditional alkaline method.

### 3.3. Cellulosic Materials

Using the technology described in the methodology, cellulose materials (composites) were obtained from fibers. Table 4 presents the parameters of the resulting cellulose composites. The highest tensile strength was demonstrated by cellulose materials obtained from shives after 40 min of EHE treatment. Even though the cellulose fibers themselves after such treatment are inferior in strength to the fibers after combined treatment (Table 3), materials based on them have higher strength properties.

Cellulose materials obtained from bast fiber after chemical and combined treatments exhibit lower strength properties. Moreover, the lowest tensile strength is observed in cellulose fiber composites after combined NaOH/EHE treatment.

**Figure 16:**
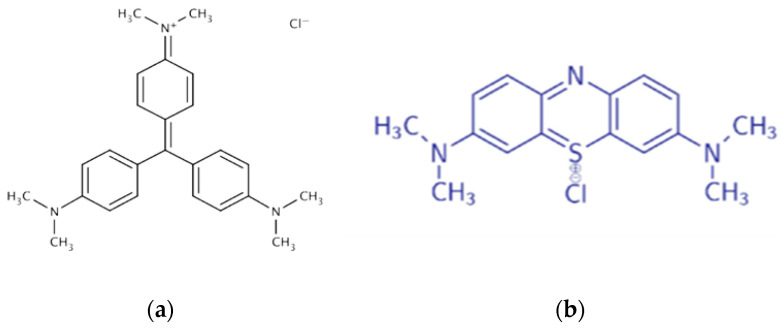
Structural formulas of dyes, methylene blue (**a**) and crystal violet (**b**).

The adsorption capacity of hemp cellulose materials will help determine their potential as a filter material. Adsorption is due to the electrostatic interaction of the amino and imino groups of the dyes (C=N) with the hydroxyl groups of cellulose, as well as hydrophobic bonds between the aromatic fragments of the dyes and the glucose rings of the polymer [20,33]. It was found that the adsorption efficiency is higher for MB than for CV (Table 5). This is due to the presence of isolated aromatic structures in MB, which provide a greater number of hydrophobic interactions with the pyranose cycles of cellulose, whereas the condensed nuclei of CV limit such bonds (Figure 16).

The best adsorption efficiency was demonstrated for a cellulose-based material after 40 min of EHE treatment. The structure of the cellulose fiber consists of amorphous and crystalline regions. It is believed that the amorphous region absorbs chemicals such as dyes and resins, while the compactness of the crystalline region hinders chemical penetration. EHE treatment likely causes defibrillation, which leads to an increase in the specific surface area of the cellulose fiber and the porosity of its structure, thereby enhancing adsorption.

The lower adsorption capacity of cellulose material based on fiber after NaOH and NaOH/EHE treatment can be attributed to changes in its chemical composition. Stevulova et al. reported that the most significant decrease in hydrophility of hemp shives was found in the case of hemp shives modified by NaOH [37]. This relates to a change in the chemical composition of hemp hurds, specifically a decrease in the average degree of cellulose polymerization and hemicellulose content.

It is known that cellulose and nanofiber cellulose exhibit high removal efficiencies, ranging from 15.0% to 90% for the former and 24% to 94% for the latter, depending on the pH, dye concentration, and adsorbent dosage. The maximum amount of adsorbed MB (qe) on cellulose and nanofiber cellulose is 94.33 and 119.04 mg/g, respectively, at an MB dye concentration of 25–150 mg/L [38]. The current research results are consistent with the reference data.

## 4. Discussion

Using electrohydraulic delignification of hemp raw materials, high-strength cellulose fibers suitable for creating composite materials were obtained. It was found that the tensile strength of cellulose fibers obtained through combined processing (NaOH/EHE) increased by 27%. In contrast, with EHE processing, the material’s strength was comparable to that of the untreated fibers, and Young’s modulus increased by 3–5 times. The developed EHE processing method for hemp raw materials eliminates the need for additional fiber separation from the shives and reduces delignification time by 2–20 times.

The strength characteristics of the fibers are generally consistent with literature data (Table 6) [22,39]. The difference in values for untreated fibers is due to differences in hemp varieties and average fiber diameter [40]. The results of tensile tests showed a pattern of changes in strength properties depending on fiber diameter. As the fiber diameter decreases, its strength increases. Morphological heterogeneity and defects present between microfibers in samples with a larger diameter reduce their overall strength. Shahzad reported that fiber strength is inversely related to fiber width, indicating that as the fiber width—and thus the number of flaws in the fiber—increases, fiber strength decreases [39]. The increased fiber strength values in Beckermann’s work are also associated with a lower fiber thickness [22].

Regarding the treated samples, those with comparable tensile strength values after EHE and combined treatment (NaOH/EHE) showed elastic modulus values that significantly exceed those of traditionally treated and untreated fibers, as well as data from literature sources.

The mechanical properties of cellulose fibers do not always determine the parameters of cellulose materials made from them. Thus, cellulose materials obtained from cellulose fiber by EHE treatment of retted fiber exhibit higher strength properties compared to materials based on cellulose fiber obtained by alkaline and combined therapy of bast fiber. It is likely that in materials obtained from shives, the cellulose fibers of the bast fiber are reinforced by those of the shives, resulting in a self-reinforcement effect that strengthens the material. Materials made from cellulose fibers after NaOH/EHE have the lowest strength properties. This is due to the embrittlement of the fiber resulting from dual chemical and EHE processing. It has been established that chemical treatment results in a decrease in the tensile strength of cellulose fibers (Table 6) [22,41].

It is evident that in cellulose materials and hemp composites, the binder (or matrix) has a significant influence on the properties of the final product. Epoxy resin, high-density polyethylene (HDPE), polyester, polyactic acid (PLA), polypropylene (PP), and poly(ε-caprolactone) (PCL) can serve as matrices for hemp composites [23,42,43,44,45]. The adhesion efficiency of epoxy and fibers also depends on the wettability of the latter. There are methods for modifying cellulose fibers to enhance their adhesive properties when combined with a binder. Previously, the authors of this work modified hemp bast fiber using the sliding arc plasma (SAP) technique [36]. It was found that adjusting the fiber in the SAP increases the wettability of its surface. The introduction of new hydrophilic groups on the surface of the fibers, combined with modifications to the surface of hemp fibers (such as splitting and increased porosity), results in enhanced wettability, which facilitates better adhesive interaction with the components of the composite material.

Cellulosic materials produced from cellulose fiber using EHE are characterized by higher strength properties, which, compared to materials based on cellulose obtained by alkaline and combined processing, increase by 10% and 50%, respectively. This is probably due to the self-reinforcement effect, where the cellulose fibers of the bast are strengthened by short cellulose fibers of the shives through close interweaving, thereby increasing the material’s strength. A similar effect has been described in [46,47] upon the introduction of nanocrystalline cellulose into a cellulose matrix. It is known that the use of graphene nanoplatelets (GNPs) as fillers in nanocomposite materials limits the movement of polymer chains by forming covalent bonds, thereby enhancing the optimal stress transmission of the GNP nanoparticles and preventing fiber pull-out in the reinforced nanocomposites [48].

Adsorption efficiency is a crucial indicator of both woven and non-woven materials. When hemp fabric is wet, the strength of the fibers increases, similar to that of cotton. Furthermore, the tensile strength of hemp fiber is eight times higher than that of cotton. Due to this, woven hemp materials can be more durable. Among non-woven materials, hemp root bast paper has potential practical applications in oil and air filters. Feng and Zhang effectively demonstrated this by comparing the oil and air filtration properties of hemp paper with commonly used automobile engine oil/air cotton paper filtration materials [24].

## 5. Conclusions

This study demonstrates the effectiveness of the EHE method in producing cellulose fibers with enhanced strength properties suitable for creating composite materials. It is shown that the type of raw material (bast or retted fiber) and its processing technology influence the material’s strength properties. EHE processing accelerates the delignification process and reduces the labor intensity of primary hemp raw material processing by eliminating the need for fiber separation. It was found that after delignification of hemp raw materials, several reflections of varying intensity appear in XRD patterns depending on the processing method, indicating increased crystallinity in samples after EHE and combined processing (NaOH/EHE).

Cellulosic materials produced from cellulose fiber using electrohydraulic processing are characterized by higher strength properties, which, compared to materials based on cellulose obtained by alkaline and combined processing, increase by 10% and 50%. The mechanical properties of cellulose materials are enhanced by a self-reinforcement effect, where bast fibers are reinforced by shive fibers, resulting in more potent mechanical properties for the entire material.

Cellulosic materials are characterized by better adsorption efficiency for methylene blue than for crystal violet. In general, the adsorption efficiency of materials made from EHE-treated fibers is higher than that of chemically treated ones. This indicator is crucial for filter materials. Therefore, the obtained results can be recommended for creating non-woven filter materials with enhanced strength characteristics.

## Figures and Tables

**Table 1:** Delignification modes of hemp raw materials.

No.	Type of Processing	Duration of Processing	Type of Raw Material
1	No treatment	-	bast fiber
2	EHE	15 min	retted fiber
3	EHE	40 min	retted fiber
4	EHE	50 min	retted fiber
5	NaOH/EHE	120 min/15 min	bast fiber
6	NaOH	120 min	bast fiber
7	NaOH	300 min	bast fiber

**Table 2:** Cellulose content after delignification.

Type of Material and Processing Parameters	Cellulose Content, wt%
Initial retted fiber	55.0
Bast fiber (NaOH, 2 h)	71.1
Retted fiber (EHE for 40 min)	60.7
Bast fiber (NaOH/EHE)	72.1

**Table 3:** Average values of deformation and strength properties of cellulose fibers.

Sample	^1^ P, N	^2^ d, mm	^3^ σ, MPa	^4^ ε, %	^5^ E, GPa
Untreated hemp fiber	7.939	0.077	190.9	1.281	16.2
	(5.561) *	(0.043)	(81.1)	(0.711)	(14.3)
Cellulose fiber (EHE for 15 min)	10.201	0.127	84.2	1.315	7.0
	(9.579)	(0.123)	(84.8)	(0.965)	(7.59)
Cellulose fiber (EHE for 40 min)	2.076	0.042	201	0.444	50.4
	(0.694)	(0.028)	(160.4)	(0.355)	(34.5)
Cellulose fiber (EHE for 50 min)	5.662	0.115	60.5	0.901	8.7
	(5.338)	(0.071)	(93.5)	(1.111)	(16.85)
Cellulose fiber (NaOH/EHE)	1.624	0.031	262	0.486	74.6
	(0.516)	(0.019)	(200.5)	(0.498)	(97.64)
Cellulose fiber (NaOH, 2 h)	1.865	0.044	126	0.730	21.8
	(1.755)	(0.019)	(88.3)	(0.378)	(18.47)
Cellulose fiber (NaOH, 5 h)	2.262	0.145	29.2	0.488	9.9
	(0.568)	0.065	(124.8)	(0.449)	(53.2)

^1^ P—breaking load, N; ^2^ d—average hemp fiber diameter, mm; ^3^ σ—tensile strength, MPa; ^4^ ε—relative elongation, %; ^5^ E—Young modulus, GPa. * Standard deviation is given in brackets.

**Table 4:** Deformation and strength properties of the cellulose materials.

Test No.	Load at Break, N	σ, MPa	ε, %	E, GPa
**Cellulose fiber-based material (retted fiber after EHE for 40 min)**				
1	25.32	4.56	3.368	0.14
2	26.29	4.74	2.877	0.16
3	17.98	3.24	2.529	0.13
4	19.98	3.60	2.684	0.13
5	28.14	5.07	4.896	0.10
6	27.76	5.00	3.209	0.16
Average	24.25	4.37	3.261	0.13
**Cellulose fiber-based material (bast fiber after NaOH/EHE)**				
1	9.44	1.70	1.774	0.10
2	15.63	2.82	2.244	0.13
3	11.79	2.12	2.112	0.10
4	11.47	2.07	1.632	0.13
5	6.14	1.11	0.818	0.14
6	10.20	1.84	1.629	0.11
Average	10.78	1.94	1.700	0.11
**Cellulose fiber-based material (bast fiber after NaOH, 2 h)**				
1	29.23	5.27	3.253	0.16
2	28.50	4.80	3.100	0.18
3	19.20	3.40	2.300	0.12
4	23.80	4.50	2.950	0.17
5	16.15	3.10	2.163	0.14
2	14.58	2.63	2.014	0.13
Average	21.91	3.95	2.630	0.15

**Table 5:** Specific surface area and adsorption efficiency for the cellulosic materials.

Treatment	Specific Surface Area, S_sp_, m^2^/g	Adsorption Efficiency, %	
		MB	CV
Cellulose fiber (EHE for 40 min)	0.305	97.45 (3.89) *	75.28 (3.09)
Cellulose fiber (NaOH/EHE)	-	83.40 (3.92)	81.72 (3.13)
Cellulose fiber (NaOH, 2 h)	-	82.55 (2.35)	73.54 (2.74)

* Standard deviation is given in brackets.

**Table 6:** Strength properties of the hemp cellulose fibers.

Treatment	Fiber Diameter, µm	Strength, MPa	Modulus, GPa	
Hemp fiber mat (circular cross-section)	67 (26) *	277 (191)	9.5 (5.7)	Shahzad [39]
Hemp fiber mat (polygonal cross-section)	67 (26)	244 (196)	8.6 (5.9)	Shahzad [39]
Untreated	26.5 (6.7)	514 (274)	24.8 (16.3)	Beckermann [22]
NaOH	21.7 (5.7)	347 (106)	20.3 (8.4)	Beckermann [22]
NaOH/Na_2_SO_4_	23.6 (5.2)	616 (260)	30.2 (11.8)	Beckermann [22]
Untreated	77 (63)	191 (81.1)	16.2 (14.3)	Current research
NaOH, 2 h	44 (19)	126 (88.3)	21.8 (18.47)	Current research
EHE for 40 min	42 (28)	201 (160.4)	50.4 (34.5)	Current research
NaOH/EHE	31 (19)	262 (200.5)	74.6 (97.64)	Current research

* Standard deviation is given in brackets.

## Data Availability

Data are contained within the article.

## Abbreviations

The following abbreviations are used in this manuscript:

EHE	Electrohydraulic Effect
MB	Methylene Blue
CV	Crystal Violet

## Appendix A

**Table A1:** Deformation and strength properties of cellulose fibers.

Test No.	P, N	d, mm	σ, MPa	ε, %	E, GPa
Untreated hemp fiber					
1	7.2	0.058 ± 0.005	272	1.992	13.7
2	5.84	0.062 ± 0.010	193	1.634	11.8
3	5.50	0.057 ± 0.010	223	1.246	17.9
4	4.98	0.050 ± 0.011	253	1.206	21.0
5	3.30	0.070 ± 0.030	168	0.550	30.5
6	6.75	0.068 ± 0.005	185	0.864	21.4
7	11.94	0.091 ± 0.017	183	1.322	13.8
8	13.59	0.120 ± 0.020	120	1.226	9.8
9	12.54	0.120 ± 0.010	111	1.236	9.0
10	7.75	0.070 ± 0.005	201	1.534	13.1
-	7.94	0.077	191	1.281	16.2
Cellulose fiber (EHE for 15 min)					
1	9.30	0.126 ± 0.250	75	1.552	4.8
2	5.78	0.070 ± 0.030	50	1.166	4.3
3	10.77	0.090 ± 0.010	169	1.268	13.3
4	19.78	0.250 ± 0.090	40	2.280	1.8
5	7.89	0.090 ± 0.020	124	0.848	14.6
6	11.76	0.110 ± 0.020	124	1.342	9.2
7	4.49	0.080 ± 0.030	89	1.258	7.1
8	17.48	0.200 ± 0.005	55	1.109	5.0
9	10.56	0.130 ± 0.015	79	1.156	6.8
10	4.20	0.120 ± 0.040	37	1.168	3.2
-	10.20	0.127	84	1.315	7.0
Cellulose fiber (EHE for 40 min)					
1	2.02	0.032 ± 0.005	257	0.516	49.8
2	2.77	0.032 ± 0.005	353	0.456	77.4
3	2.03	0.040 ± 0.010	162	0.238	68.1
4	2.01	0.050 ± 0.020	103	0.201	51.2
5	2.12	0.070 ± 0.005	55	0.398	13.8
6	2.03	0.035 ± 0.010	211	0.251	84.1
7	2.11	0.030 ± 0.010	298	0.798	37.3
8	2.02	0.060 ± 0.005	71	0.638	11.1
9	2.15	0.045 ± 0.020	135	0.514	26.3
10	1.50	0.023 ± 0.030	361	0.425	84.9
-	2.08	0.042	201	0.444	50.4
Cellulose fiber (EHE for 50 min)					
1	11.00	0.130 ± 0.050	83	2.012	4.1
2	2.94	0.086 ± 0.010	51	0.200	25.5
3	6.36	0.140 ± 0.040	41	0.928	4.4
4	10.08	0.186 ± 0.040	37	1.026	3.6
5	9.06	0.110 ± 0.020	95	0.780	12.2
6	2.08	0.103 ± 0.010	25	0.478	5.2
7	2.51	0.115 ± 0.010	24	0.748	3.2
8	5.56	0.100 ± 0.010	71	1.392	5.1
9	4.36	0.060 ± 0.005	154	0.786	19.6
10	2.67	0.118 ± 0.020	24	0.660	3.6
-	5.66	0.115	61	0.901	8.7
Cellulose fiber (NaOH/EHE)					
1	1.45	0.020 ± 0.020	462	0.844	54.7
2	1.24	0.023 ± 0.010	291	0.190	153.2
3	1.19	0.018 ± 0.005	452	0.262	172.5
4	1.34	0.027 ± 0.005	241	0.242	99.6
5	1.60	0.030 ± 0.020	226	0.512	44.1
6	2.07	0.043 ± 0.015	142	0.984	14.4
7	2.05	0.037 ± 0.010	194	0.352	51.1
8	2.14	0.028 ± 0.030	340	0.422	80.6
9	1.54	0.050 ± 0.005	78	0.764	10.2
10	1.62	0.033 ± 0.010	189	0.290	65.2
-	1.62	0.031	262	0.486	74.6
Cellulose fiber (NaOH, 5 h)					
1	1.21	0.100 ± 0.005	154	0.244	63.1
2	2.68	0.200 ± 0.010	9	0.936	0.9
3	2.59	0.120 ± 0.010	23	0.536	4.3
4	1.95	0.130 ± 0.005	15	0.222	6.8
5	2.83	0.200	9	0.786	1.1
6	2.80	0.120 ± 0.010	25	0.536	4.7
7	2.32	0.150 ± 0.005	13	0.478	2.7
8	2.01	0.110	21	0.322	6.5
9	1.45	0.110 ± 0.020	15	0.198	7.6
10	2.78	0.210 ± 0.030	8	0.617	1.3
-	2.26	0.145	29	0.488	9.9
Cellulose fiber (NaOH, 2 h)					
1	2.37	0.056 ± 0.010	96	0.654	14.7
2	3.62	0.063 ± 0.030	116	1.032	11.2
3	1.51	0.030 ± 0.010	214	0.896	23.9
4	1.45	0.042 ± 0.050	107	1.108	9.7
5	1.47	0.050 ± 0.030	75	0.186	40.3
6	1.21	0.040 ± 0.020	96	0.264	36.4
7	1.12	0.030 ± 0.015	158	0.756	20.9
8	2.73	0.050 ± 0.020	139	1.010	13.8
9	1.62	0.036 ± 0.012	159	1.104	14.4
10	1.55	0.045 ± 0.010	97	0.294	33.0
-	1.87	0.044	126	0.730	21.8
